# Protective Role of Rosmarinic Acid–Enriched *Elsholtzia kachinensis* Extract Against Oxidative and Inflammatory Stress in Human Trophoblasts Through the miR‐138‐5p/FOXC1 Pathway

**DOI:** 10.1155/bri/6626588

**Published:** 2026-05-21

**Authors:** Wittaya Chaiwangyen, Orawan Khantamat, Amnart Onsa-ard, Napapan Kangwan, Wachiraporn Tipsuwan, Angkana Songkrao, Apirak Katasai, Apisit Phngam, Piyawan Nuntaboon, Ramita Sangprachum, Pilaiporn Thippraphan, Francisco Lázaro Pereira de Sousa

**Affiliations:** ^1^ Division of Biochemistry, School of Medical Sciences, University of Phayao, Phayao, 56000, Thailand, up.ac.th; ^2^ Department of Biochemistry, Faculty of Medicine, Chiang Mai University, Chiang Mai, 50200, Thailand, cmu.ac.th; ^3^ Division of Physiology, School of Medical Sciences, University of Phayao, Phayao, 56000, Thailand, up.ac.th; ^4^ Clinical Chemistry Laboratory, Chiang Rai Prachanukroh Hospital, Chiang Rai, 57000, Thailand; ^5^ Department of Gynecology and Obstetrics, UNILUS (Centro Universitário Lusíada), Santos, 11050-071, Brazil

**Keywords:** *Elsholtzia kachinensis* Prain, FOXC1, inflammation, miR-138-5p, oxidative stress, trophoblast

## Abstract

Inflammation‐induced dysregulation of microRNAs, particularly miR‐138‐5p, compromises trophoblast cell function and may contribute to pregnancy‐related complications. While *Elsholtzia* species have been reported to possess therapeutic properties, the anti‐inflammatory effects of *Elsholtzia kachinensis* Prain (EKP) in trophoblast cells have not been explored. In this study, a 70% ethanolic extract of EKP was fractionated into hexane (HEX), dichloromethane (DCM), ethyl acetate (ETAC), and water (WT) fractions. Each fraction was subjected to phytochemical profiling, antioxidant assessment, and bioactive compound identification by GC–MS and high‐performance liquid chromatography (HPLC). Among them, the ETAC fraction (EKP‐ETAC) showed the highest antioxidant activity, reflected by the lowest IC_50_ values in ABTS and DPPH assays, and contained the greatest levels of phenolic and flavonoid compounds. Nontoxic concentrations were determined by an MTT assay. Pretreatment with EKP‐ETAC alleviated lipopolysaccharide (LPS)‐induced impairment of proliferation, migration, and invasion in HTR‐8/SVneo trophoblast cells, while reducing apoptosis, intracellular ROS generation, and inflammatory cytokine expression. Mechanistic analysis revealed that EKP‐ETAC suppressed LPS‐induced upregulation of miR‐138‐5p and restored the expression of FOXC1. The dual luciferase reporter assay confirmed that FOXC1 is a direct target of miR‐138‐5p, mediating the effects of EKP‐ETAC. LC–MS and HPLC analyses identified rosmarinic acid as the predominant bioactive component. These findings suggest that rosmarinic acid‐enriched EKP‐ETAC exerts protective effects on trophoblast cells by mitigating oxidative stress and inflammation through the miR‐138‐5p/FOXC1 axis.

## 1. Introduction

Inflammation in the placenta during pregnancy can occur from various causes, including microbial infection and nonmicrobial infections such as oxidative stress and environmental factors [1]. Such inflammation has been associated with pregnancy complications, including preterm birth, miscarriage, neurodevelopmental disorders, and preeclampsia [2]. Trophoblast cells establish the boundary between the fetus and the maternal interface, supporting gas exchange and hormone production through processes such as trophoblast invasion, migration, proliferation, and the remodeling of uterine spiral arteries [3]. Insufficient trophoblast cell function could lead to reduced blood flow into the intervillous space, thereby contributing to placenta development disorder and pregnancy complications [4]. Lipopolysaccharide (LPS)‐induced inflammation through increased production of proinflammatory cytokines can suppress trophoblast migration and invasion and induce apoptosis.

MicroRNAs (miRNAs) are single‐stranded, endogenous, and short noncoding RNAs with approximately 22 nucleotides that are pivotal in the post‐transcriptional regulation of gene expression [5]. Currently, miRNAs are widely recognized for their role in regulating cellular development and functions across different cell types, including throughout pregnancy [6]. Dysregulated miRNAs contribute to pathological conditions through upregulation or downregulation, which is implicated in diseases, including pregnancy disorders [7]. Numerous miRNAs promote inflammation, such as miR‐21, miR‐23a, miR‐25, miR‐27a, miR‐27c, miR‐29b, miR‐34a, miR‐34c, miR‐92a, miR‐126, miR‐138‐5p, miR‐155, and Let‐7a [8].

miR‐138‐5p is a tumor‐suppressor miRNA known to inhibit epithelial–mesenchymal transition (EMT), cell proliferation, and tumor metastasis in various cancers [9]. Recent studies suggest that miR‐138‐5p also plays a vital function in controlling inflammation in trophoblasts. However, in gestational diabetes mellitus (GDM), miR‐138‐5p levels are inversely correlated with placental weight and are associated with decreased trophoblast cell proliferation, migration, and invasion [10]. Specifically, miR‐138‐5p has been shown to significantly inhibit the migration and proliferation of HTR‐8/SVneo trophoblasts by targeting *TBL1X* and *EZH2* mRNA [10, 11].

*Elsholtzia* of the Lamiaceae family includes at least 42 species reported for their substantial polyphenols, flavonoids, and glycosides [12]. *Elsholtzia* species have historically been used in Chinese folk medicine to treat inflammatory conditions such as colds, pneumonia, and fever [13]. Their ethnopharmacological activities demonstrate that members of the *Elsholtzia* genus possess antiviral, antibacterial, antioxidant, and anti‐inflammatory properties, mediated through the inhibition of nitric oxide (NO), IL‐1β, IL‐6, TNF‐α, NF‐κB, and MAPKs [13].

*Elsholtzia kachinensis* Prain (EKP), also known as Pak Luean in Thailand, is a species predominantly distributed in Asia, including Myanmar, Vietnam, and China [14]. Its edible leaves are commonly used in Northern Thai cuisine [15]. In China, EKP is an annual herb traditionally used in Guangxi, Jiangxi, Sichuan, Hubei, Yunnan, Guangdong, Hunan, and Guizhou Provinces as both a vegetable and a folk herbal medicine for treating inflammation‐related conditions, including fever, edema, and pain [16, 17]. It is also traditionally applied for treating diarrhea and bloating [14, 18]. Ethnopharmacological studies have documented its anti‐inflammatory property via NO, a key inflammatory mediator, as well as antibacterial, antiviral, and insecticidal properties [16, 17, 19]. Chemical analysis has identified octenyl acetate, carvone, dehydroelsholtzia ketone, and other compounds as major components of its essential oil [17, 19].

While substantial study has been conducted on the pharmacological activities and phytochemical composition of various *Elsholtzia* species, such as *E. splendens*, *E. ciliata*, *E. blanda*, and *E. bodinieri*, studies on the anti‐inflammatory mechanisms of *E. kachinensis* remain limited [12]. Despite its well‐documented traditional use in treating inflammation‐related conditions, the molecular mechanisms underlying these anti‐inflammatory functions have not been entirely understood. This study aims to investigate the biological activities and phytochemical profile of EKP, notably its anti‐inflammatory and antioxidant effects in trophoblast cells, which are consistent with its traditional usage in Chinese folk medicine. Based on ethnopharmacological findings, we propose that EKP extracts may attenuate inflammatory responses simultaneously, improving trophoblast cell functions. Accordingly, this research examines the protective effects of EKP extract against LPS‐induced inflammation in trophoblast cells, with particular emphasis on miR‐138‐5p regulatory pathways.

## 2. Materials and Methods

### 2.1. Plant Extraction

EKP was gathered in Chiang Rai Province, Thailand, and a voucher specimen no. 0023398 was archived at the Herbarium, Faculty of Pharmacy, Chiang Mai University, Thailand. The plant materials were dried at 40°C for 96 h. Following drying, the material was extracted with 70% ethanol for 24 h. The extract underwent fractionation with several solvents, including hexane (HEX), dichloromethane (DCM), ethyl acetate (ETAC), and water (WT). The solvents were evaporated using a rotary evaporator, while freeze‐drying was employed to obtain the water fraction. The yields of EKP extracts were recorded, and the extracts were kept at −20°C for later investigation. Yield percentages were determined by dividing the extract weight by the dried material weight and multiplying by 100.

### 2.2. Total Phenolic Content (TPC)

The TPC assessment for all five extracts utilized the Folin–Ciocalteu method. Specifically, each sample was combined with sodium carbonate and a Folin–Ciocalteu solution. After incubating for 30 min, the absorbance measurements were recorded at the wavelength 765 nm. Results were quantified as gallic acid equivalents (GAE), with final values reported as milligrams of GAE per gram of dry extract weight.

### 2.3. Total Flavonoid Content (TFC)

To evaluate the TFC in the five extracts, an aluminum chloride‐based colorimetric assay was employed. In this procedure, each sample was mixed with a sodium nitrite solution and left at room temperature for 5 min. This was followed by the addition of aluminum chloride and sodium hydroxide solutions. After a 10‐min incubation period, the absorbance readings were measured at a wavelength of 510 nm. TFC values were expressed as milligrams of catechin equivalents (CE) per gram of dried extract.

### 2.4. Liquid Chromatography–Mass Spectrometry (LC–MS)

The ETAC fraction of EKP underwent chemical composition analysis using an Agilent 1290 Infinity LC system connected to an Agilent 6540 Series QTOF‐MS equipped with a diode‐array detector and ESI source. The high‐performance liquid chromatography (HPLC) column employed for analysis was Agilent Poroshell 120 EC‐C18 with dimensions of 4.5 mm x 150 mm  and particle size of 2.7 μm. The analysis employed water and acetonitrile mobile phases, each containing 0.1% formic acid, flowing at 200 μL/min. MS spectra were collected in negative ion mode. Compound identification involved comparing mass spectral data of unknown peaks and retention times against reference standards or published literature information.

### 2.5. HPLC

Chemical composition analysis was conducted on a Shimadzu LC‐20A HPLC system (Kyoto, Japan) with a UV–Vis diode array detector, operating at a wavelength of 280 nm for quantification. The analysis utilized an Agilent ZORBAX Eclipse Plus C18 column (4.6 mm × 150 mm,  5 μm) (Santa Clara, CA, USA), maintained at 30°C. Each injection volume was 10 μL. The mobile phase consisted of solvent A (0.1% trifluoroacetic acid in water) and solvent B (0.1% trifluoroacetic acid in methanol). All samples and standards, including caffeic acid, chlorogenic acid, luteolin, and rosmarinic acid, were analyzed using the same gradient program over 10 min at a flow rate of 1.0 mL/min. Rosmarinic acid content was quantified using a rosmarinic acid reference standard.

### 2.6. DPPH Radical Scavenging Activity

The effectiveness of EKP as a donor of hydrogen atoms or electrons was evaluated by measuring the reduction of the DPPH radical. Different concentrations of Trolox as standard and EKP extracts were mixed with DPPH solution and incubated for 30 min at room temperature under dark conditions. Then, the absorbance at 515 nm was recorded. The scavenging activity was calculated as a percentage using the formula:

(1) Percentage of DPPH radical scavenging activity=A0−AsA0×100.

In this calculation, A_0_ denotes the control absorbance, while *A*_*s*_ indicates the samples absorbance. IC_50_ values were obtained by graphing the percentage inhibition against different extract concentrations.

### 2.7. ABTS Radical Scavenging Activity

The effectiveness of EKP in neutralizing free radicals was determined using the ABTS^•+^ radical cation decolorization assay. The ABTS^•+^ solution was prepared by combining 7.5 mM ABTS solution with 2.45 mM potassium persulfate, then stored in darkness at room temperature for 12–16 h. Prior to testing, the ABTS^•+^ working solution was prepared by adjusting its concentration with deionized water to obtain an absorbance of 0.700 ± 0.02 at 734 nm. The extracts or Trolox standard were then added to this solution, and absorbance measurements were taken at 734 nm after 6 min. The radical scavenging activity was calculated using the equation:

(2) Percentage of ABTS radical scavenging activity=1−AsA0×100.

In this calculation, *A*_0_ denotes the control absorbance, while *A*_*s*_ indicates the sample absorbance. IC_50_ values were determined by plotting inhibition percentages against various extract concentrations.

### 2.8. Cell Line and Treatment

The HTR‐8/SVneo trophoblast cell line was obtained as a gift from Professor Charles H. Graham (Kingston, Canada) and cultured in RPMI medium (Cytiva, Massachusetts, United States) enriched with 1% penicillin/streptomycin (Thermo Fisher Scientific, Dreieich, Germany) and 10% heat‐inactivated fetal bovine serum (FBS) (Cytiva, Massachusetts, United States) at 37°C in a 5% CO_2_ atmosphere.

### 2.9. Cell Viability

The cytotoxicity of EKP‐ETAC was evaluated using the MTT assay (Sigma‐Aldrich, Darmstadt, Germany). HTR‐8/SVneo cells were plated in 96‐well plates at a density of 5 × 10^3^ cells per well. Following exposure to different concentrations of EKP for periods ranging from 24 to 72 h, the cells were treated with 100 μL of MTT solution and incubated for 4 h at 37°C. After incubation, an equal volume of DMSO was added to dissolve the formed formazan crystals. The absorbance was then measured at 570 nm using a microplate reader.

### 2.10. Proliferation Assay

HTR‐8/SVneo cell proliferation was assessed using a BrdU ELISA assay (Sigma‐Aldrich, Darmstadt, Germany). HTR‐8/SVneo cells were seeded in 96‐well plates at 5 × 10^3^ cells per well and pretreated with EKP‐ETAC at 10 and 40 μg/mL concentrations for 24 h before being exposed to 1 μg/mL LPS for periods ranging from 24 to 72 h. Following this treatment, BrdU was added for 2 h and underwent fixation. Subsequently, cells were treated with a peroxidase‐conjugated anti‐BrdU monoclonal antibody. After a washing step, substrate solution was added, and the reaction was terminated using 1 M sulfuric acid solution. Absorbance was recorded at 450/690 nm with a microplate reader.

### 2.11. Transwell Migration and Matrigel Invasion Assay

HTR‐8/SVneo cell migration and invasion evaluations were conducted using 24‐well inserts (8‐μm pore size) (Sigma‐Aldrich, Darmstadt, Germany). Cells (1 × 10^5^) were initially treated with EKP‐ETAC and subsequently exposed to LPS in a serum‐free medium. For invasion testing, cells were applied to Matrigel‐coated membranes (Corning, AZ, USA), while cell migration assessment used uncoated inserts. The lower chamber contained medium with 20% FBS as a chemoattractant. Cells that traversed to the lower membrane surface were fixed using cold ethanol and stained with crystal violet. Following decolorization with acetic acid, absorbance was measured at 570 nm.

### 2.12. Intracellular Reactive Oxygen Species (ROS) Assay

To assess how EKP‐ETAC inhibits intracellular ROS, we utilized the fluorometric intracellular ROS assay kit (Sigma, St. Louis, MO, USA). Cells were seeded in black 96‐well plates at a density of 5 × 10^3^ cells per well and allowed to attach overnight. The following day, EKP‐ETAC treatment was applied to the cells. Following a 24 h incubation period at 37°C, oxidative stress was induced by adding 100 μM H_2_O_2_ for 1 h. Subsequently, the Master Reaction Mix was introduced to the cells, and fluorescence intensity measurements were taken using a microplate reader with excitation and emission wavelengths set at 490 and 525 nm, respectively.

### 2.13. Apoptosis Assay

Apoptosis analysis was conducted using the Annexin V‐FITC Apoptosis Detection Kit (BD, New Jersey, USA). The cells underwent treatment with EKP‐ETAC for 24 h before exposure to LPS for an additional 24 h. Following treatment, the cell suspensions were incubated at room temperature with Annexin V and PI in dark conditions for 15 min. Flow cytometry (BD FACSLyric, Becton Dickinson, Waltham, MA, USA) was then used to quantify apoptotic cells.

### 2.14. Cell Cycle Analysis

Cell cycle analysis was performed using the FxCycle PI/RNase Staining kit (Thermo Fisher Scientific, Dreieich, Germany). Cells were initially treated with EKP‐ETAC for 24 h before exposure to LPS for an additional 24 h. After harvesting, cells underwent fixation with 70% ethanol and were subsequently washed with PBS. The cell pellet was then treated with staining solution and kept in darkness at room temperature for 15 min. Flow cytometry was employed for cell cycle analysis.

### 2.15. RNA Isolation

RNA was extracted from control cells and those with EKP‐ETAC followed by LPS exposure using TRIzol reagent (Invitrogen, Darmstadt, Germany) according to the manufacturer’s instructions. RNA concentration measurements were performed on a NanoDrop spectrophotometer (PeqLab Biotechnologies GmbH, Erlangen, Germany). Samples showing an A260/A280 ratio greater than 1.8 were preserved at −80°C for subsequent analysis.

### 2.16. Quantification of miRNA Expression

Quantitative reverse transcription PCR (qRT‐PCR) was conducted using the QIAquant 96 system (Qiagen, Düsseldorf, Germany), preceded by the conversion of RNA to cDNA (Qiagen, Düsseldorf, Germany). miRNA analysis employed the miRCURY LNA SYBR Green PCR Kit (Qiagen, Düsseldorf, Germany). The level of miR‐138a‐5p was determined using the 2^−ΔΔCt^ method, with SNORD48 serving as the reference control.

### 2.17. Target of miRNA Prediction

Target prediction for miRNA was performed utilizing an online database, TargetScan (https://www.targetscan.org/vert_71/).

### 2.18. Assessment of Inflammatory Cytokines and miRNA Targeting via qRT‐PCR

The assessment of *IL-1β*, *IL-6*, and *TNF-α* gene expression involved first converting RNA to cDNA using the Reverse Transcription Kit (Thermo Fisher Scientific, MA, USA). Subsequently, the SYBR Green qPCR Master Mix (Thermo Fisher Scientific, MA, USA) was employed for quantitative PCR analysis (QIAquant 96, Qiagen). Expression levels were measured using the 2^−ΔΔCt^ method with GAPDH serving as the housekeeping gene for normalization. The specific primers used for this analysis were detailed in Table 1 [20].

**TABLE 1:** Specific primers.

Primers	Forward sequence	Reverse sequence
GAPDH	5ʹ‐AGC​CAC​ATC​GCT​CAG​ACA​C‐3ʹ	5ʹ‐GCC​CAA​TAC​GAC​CAA​ATC​C‐3ʹ
IL‐6	5ʹ‐AGA​CAG​CCA​CTC​ACC​TCT​TCA​G‐3ʹ	5ʹ‐TTC​TGC​CAG​TGC​CTC​TTT​GCT​G‐3ʹ
TNF‐α	5ʹ‐CCC​AGG​CAG​TCA​GAT​CAT​CTT​C‐3ʹ	5ʹ‐AGC​TGC​CCC​TCA​GCT​TGA‐3ʹ
IL‐1β	5ʹ‐CCA​CAG​ACC​TTC​CAG​GAG​AAT​G‐3ʹ	5ʹ‐GTG​CAG​TTC​AGT​GAT​CGT​ACA​GG‐3ʹ
FOXC1	5ʹ‐CCA​CTC​GGT​GCG​GGA​AAT​G‐3ʹ	5ʹ‐ACG​TGC​GGT​ACA​GAG​ACT​GA‐3ʹ

### 2.19. Dual Luciferase Reporter Assay

The wild‐type (WT) forkhead box c1 (FOXC1) 3′‐UTR sequence (5′‐UCC​AAA​AAU​UCA​GCU​CAC​CAG​CA‐3′) and mutant (MUT) FOXC1 3′‐UTR sequence (5′‐UCC​AAA​AAU​UCA​GCU​GUG​GUC​GA‐3′) were constructed and inserted into the pmirGLO dual luciferase miRNA target expression vector (Promega, Wisconsin, USA) as the FOXC1‐WT or FOXC1‐MUT luciferase reporter plasmid. HTR‐8/SVneo cells were co‐transfected with 20 nM miR‐138‐5p mimic or negative control (NC) mimic and 0.5 μg/mL FOXC1‐WT and FOXC1‐MUT using DharmaFECT Duo transfection reagent (Horizon Discovery, Cambridge, UK) for 48 h. The dual luciferase activities of Firefly and Renilla were measured and normalized with Renilla luciferase activity using a dual luciferase reporter assay system according to the manufacturer’s instructions (Promega, Wisconsin, USA). The multifunctional microplate reader was used to measure luminescence intensity (BMG Labtech, Ortenberg, Germany).

### 2.20. Statistical Analysis

All experiments were performed in three independent replicates, each with triplicate technical measurements. Statistical analyses were conducted using GraphPad Prism Version 8.0 (GraphPad Software, San Diego, CA, USA) on data from these three independent experiments. Results are expressed as mean ± standard deviation (SD). Statistical significance was determined by one‐way ANOVA followed by Tukey’s multiple comparison test. Significance levels were set at ^∗^ *p* < 0.05, ^∗∗^ *p* < 0.01, and ^∗∗∗^ *p* < 0.001.

## 3. Results

### 3.1. Extraction and Phytochemical Analysis

The EKP extract was prepared by extracting 200 g of plant material with 70% ethanol, yielding 17.5% of ethanol crude extract (EKP‐ECE). This extract was subsequently partitioned into four fractions: HEX, DCM, ETAC, and WT, resulting in the following yields (as shown in Table 2): EKP‐HEX (0.44%), EKP‐DCM (0.25%), EKP‐ETAC (0.65%), and EKP‐WT (3.32%).

**TABLE 2:** The percentage yield of *E. kachinensis* Prain extracts.

Fraction	Weight (g)	% yield
Ethanol crude extract (EKP‐ECE)	35.08	17.50
Hexane fraction (EKP‐HEX)	0.88	0.44
Dichloromethane fraction (EKP‐DCM)	0.50	0.25
Ethyl acetate fraction (EKP‐ETAC)	1.30	0.65
Water fraction (EKP‐WT)	6.63	3.32

The phytochemical analysis focused on determining the TFC and TPC in the fractions. The TFC values ranged from 82.14 to 440.09 mg CE/g extract, with the lowest content found in EKP‐HEX and the highest in EKP‐ETAC. Similarly, the TPC ranged from 128.50 mg GAE/g extract (EKP‐HEX) to 667.22 mg GAE/g extract (EKP‐ETAC), as summarized in Table 3. Our findings indicate that the EKP‐ETAC fraction had the highest TFC and TPC among all fractions.

**TABLE 3:** TFC and TPC in *E. kachinensis* Prain extracts.

Extracts	TFC (mg CE/g extract)	TPC (mg GAE/g extract)
Ethanol crude extract (EKP‐ECE)	179.41 ± 6.8	216.40 ± 3.9
Hexane fraction (EKP‐HEX)	82.14 ± 3.3	128.50 ± 2.8
Dichloromethane fraction (EKP‐DCM)	105.84 ± 1.9	145.29 ± 2.6
Ethyl acetate fraction (EKP‐ETAC)	440.09 ± 12.6	667.22 ± 4.4
Water fraction (EKP‐WT)	119.20 ± 2.9	155.90 ± 2.1

### 3.2. Antioxidant Activities of EKP Extracts

The antioxidant potential of EKP fractions was assessed through ABTS and DPPH radical scavenging assays. The IC_50_ values, which reflect the concentration required to inhibit 50% of the free radicals, are presented in Table 4. EKP‐ETAC demonstrated the strongest antioxidant activity, with IC_50_ values of 3.51 ± 0.10 μg/mL (ABTS assay) and 12.77 ± 0.14 μg/mL (DPPH assay). These values were comparable to those of the standard antioxidants ascorbic acid (2.53 ± 0.01 μg/mL and 6.65 ± 0.15 μg/mL, respectively) and Trolox (2.98 ± 0.03 μg/mL and 10.32 ± 0.02 μg/mL, respectively). The weakest antioxidant activities were observed in the EKP‐WT for the ABTS assay (13.89 ± 0.20 μg/mL) and in the EKP‐HEX for the DPPH assay (60.04 ± 1.74 μg/mL). These findings are consistent with the TFC and TPC measurements, as EKP‐ETAC, which exhibited the highest TFC and TPC, also demonstrated the strongest antioxidant activity. Consequently, EKP‐ETAC was selected for further studies on its biological activities.

**TABLE 4:** Antioxidant activities of *E. kachinensis* Prain extracts.

Extracts	ABTS IC_50_ (μg/mL)	DPPH IC_50_ (μg/mL)
Ethanol crude extract (EKP‐ECE)	8.90 ± 0.07	32.72 ± 0.51
Hexane fraction (EKP‐HEX)	12.57 ± 0.43	60.04 ± 1.74
Dichloromethane fraction (EKP‐DCM)	12.74 ± 0.04	50.66 ± 0.59
Ethyl acetate fraction (EKP‐ETAC)	3.51 ± 0.10	12.77 ± 0.14
Water fraction (EKP‐WT)	13.89 ± 0.20	42.24 ± 0.60
Ascorbic acid	2.53 ± 0.01	6.65 ± 0.15
Trolox	2.98 ± 0.03	10.32 ± 0.02

### 3.3. Effect of EKP‐ETAC on HTR‐8/SVneo Cell Viability

**FIGURE 1:**
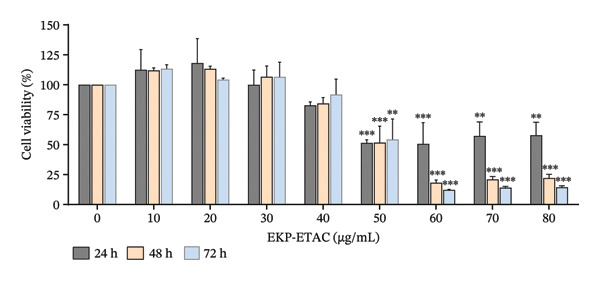
Cell viability of HTR‐8/SVneo cells upon EKP‐ETAC treatment. ^∗∗^ *p* < 0.01, ^∗∗∗^ *p* < 0.001.

The cytotoxicity of EKP‐ETAC on HTR‐8/SVneo cells was assessed using the MTT assay. Cells were exposed to EKP‐ETAC at concentrations ranging from 0 to 80 μg/mL for 24–72 h. As shown in Figure 1, treatments exceeding 50 μg/mL significantly decreased cell viability throughout the time course. Based on these findings, EKP‐ETAC concentrations of 10  and 40 μg/mL were selected for subsequent studies, as these concentrations were determined to be nontoxic.

### 3.4. Effect of EKP‐ETAC on Cell Proliferation in LPS‐Induced HTR‐8/SVneo Cells

**FIGURE 2:**
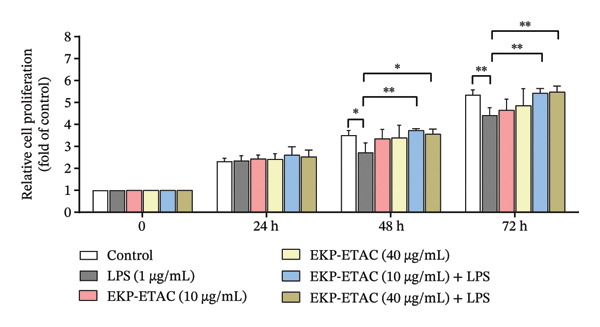
HTR‐8/SVneo cell proliferation under various treatment conditions over 24–72 h. Cells were pretreated with EKP‐ETAC followed by LPS exposure or treated with LPS or EKP‐ETAC alone. Cell proliferation was assessed using the BrdU incorporation assay. ^∗^ *p* < 0.05, ^∗∗^ *p* < 0.01.

The impact of EKP‐ETAC on HTR‐8/SVneo cell proliferation in response to LPS was evaluated using a BrdU incorporation assay. Cells were pretreated with EKP‐ETAC at concentrations of 10 or 40 μg/mL and subsequently co‐treated with or without LPS. The results revealed that LPS treatment alone significantly reduced cell proliferation compared to the control group at 48–72 h (Figure 2). However, pretreatment with EKP‐ETAC effectively protected HTR‐8/SVneo cells from LPS‐induced suppression of proliferation. These findings suggest that EKP‐ETAC has the potential to restore cell proliferation under inflammatory conditions.

### 3.5. Anti‐Inflammatory Effect of EKP‐ETAC on LPS‐Induced HTR‐8/SVneo Cells

**FIGURE 3:**
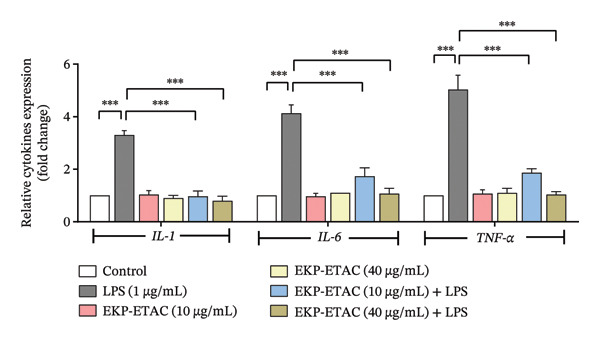
The expression of *IL-1*, *IL-6*, and *TNF-α* in HTR‐8/SVneo cells. Cells were pretreated with EKP‐ETAC and followed by LPS exposure or treated with LPS and EKP‐ETAC alone for 24 h. ^∗∗∗^ *p* < 0.001.

The anti‐inflammatory potential of EKP‐ETAC was assessed by measuring the expression levels of proinflammatory cytokines gene *IL-1*, *IL-6*, and *TNF-*α. LPS treatment significantly increased cytokine expression in HTR‐8/SVneo cells compared to untreated controls (Figure 3). However, pretreatment with EKP‐ETAC at concentrations of 10 and 40 μg/mL, followed by LPS exposure, significantly reduced the expression of all measured cytokines compared to the LPS‐only group. Notably, EKP‐ETAC treatment alone did not affect cytokine expression. These findings indicate that EKP‐ETAC exerts protective anti‐inflammatory effects in LPS‐induced trophoblast cells.

### 3.6. Effect of EKP‐ETAC on Cell Migration and Invasion in LPS‐Induced HTR‐8/SVneo Cells

The effect of EKP‐ETAC on LPS‐induced suppression of trophoblast cell migration and invasion, both critical processes for a successful pregnancy, was further investigated. The results revealed that LPS reduced HTR‐8/SVneo cell migration by 35% and invasion by 25% compared to the control. However, pretreatment with EKP‐ETAC prior to LPS stimulation significantly restored these functions to levels comparable to the control group (Figure 4).

FIGURE 4HTR‐8/SVneo cell migration (a) and invasion (b). Cells were pretreated with EKP‐ETAC and followed by LPS exposure or treated with LPS and EKP‐ETAC alone for 24 h. ^∗^ *p* < 0.05, ^∗∗^ *p* < 0.01, ^∗∗∗^ *p* < 0.001.

**Figure:**
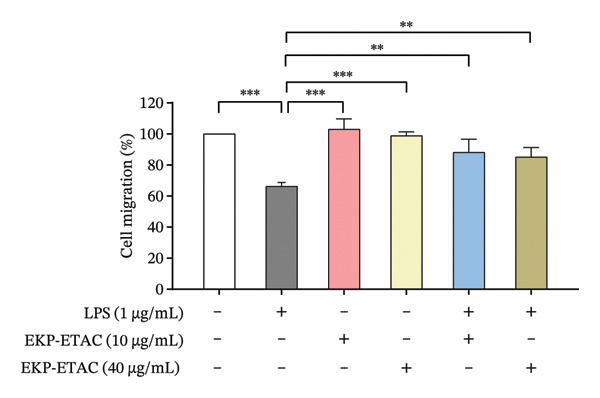
(a)

**Figure:**
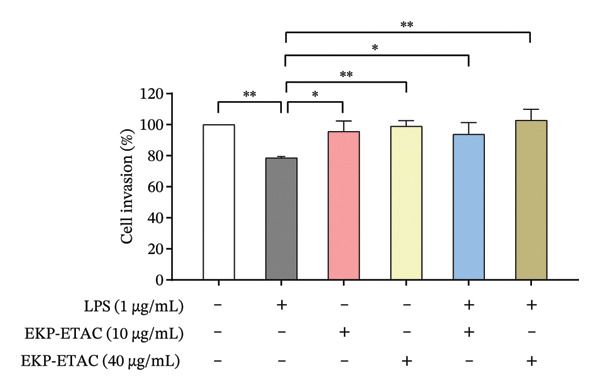
(b)

### 3.7. Effect of EKP‐ETAC on LPS‐Induced Apoptosis in HTR‐8/SVneo Cells

This experiment investigated the protective effect of EKP‐ETAC on LPS‐induced apoptosis in trophoblast cells using flow cytometry. The results demonstrated that LPS significantly induced both apoptosis and necrosis in HTR‐8/SVneo cells. However, preincubation with EKP‐ETAC at concentrations of 10 and 40 μg/mL effectively mitigated these effects, restoring cell viability and reducing LPS‐induced apoptosis and necrosis (Figure 5). These findings suggest that EKP‐ETAC exerts protective effects against apoptosis and necrosis in trophoblast cells.

FIGURE 5Apoptosis and necrosis of HTR‐8/SVneo cells. Cells were preincubated with EKP‐ETAC and then stimulated with LPS or treated with LPS and EKP‐ETAC alone for 24 h. Following this, cell apoptosis and necrosis were assessed using flow cytometry (a), and the total apoptosis and necrosis were calculated and compared to the control (b). ^∗^ *p* < 0.05, ^∗∗^ *p* < 0.01.

**Figure:**
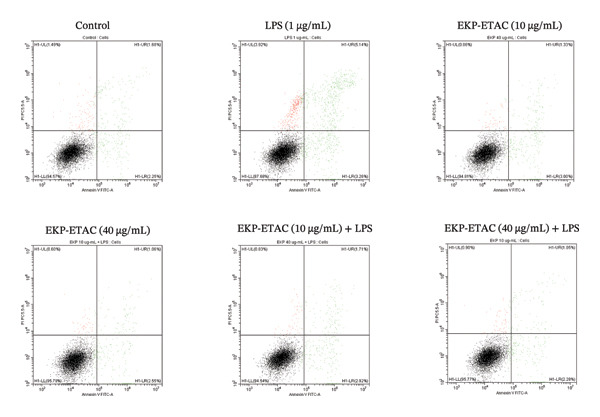
(a)

**Figure:**
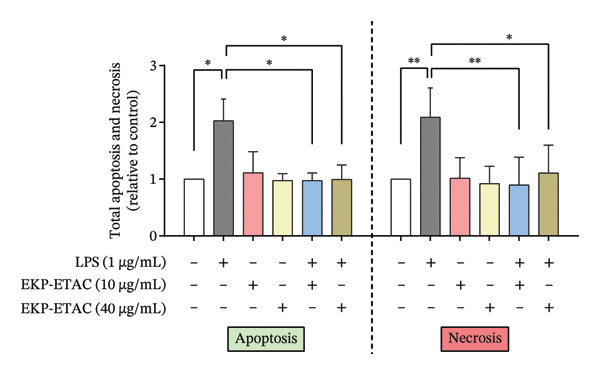
(b)

### 3.8. Effect of EKP‐ETAC on the Cell Cycle of LPS‐Induced HTR‐8/SVneo Cells

The impact of EKP‐ETAC on the LPS‐induced cell cycle in HTR‐8/SVneo cells was further examined by analyzing DNA content across the sub‐G1, G0/G1, S, and G2/M phases using flow cytometry (Figure 6). LPS treatment significantly increased the proportion of cells in the sub‐G1 phase compared to the control, indicating the occurrence of apoptosis. It also tended to induce cell cycle arrest in the G0/G1 phase, accompanied by a slight reduction in the S and G2/M phases. In contrast, pretreatment with EKP‐ETAC at concentrations of 10 and 40 μg/mL reduced apoptosis (sub‐G1 phase) and alleviated cell cycle arrest in LPS‐induced HTR‐8/SVneo cells. These findings suggest that EKP‐ETAC may protect trophoblast cells from LPS‐induced apoptosis.

FIGURE 6Cell cycle analysis in HTR‐8/SVneo cells. Cells were preincubated with EKP‐ETAC and then stimulated with LPS or treated with LPS and EKP‐ETAC alone for 24 h. The cell cycle was determined using flow cytometry (a), and the cell cycle distribution was calculated and compared to the control (b). ^∗^ *p* < 0.05, ^∗∗∗^ *p* < 0.001.

**Figure:**
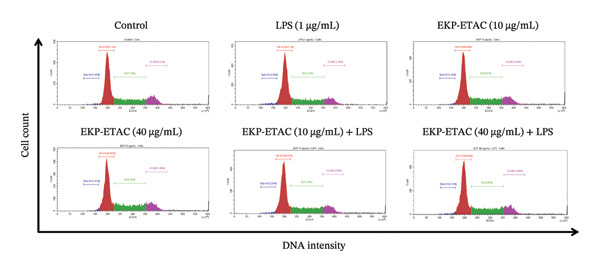
(a)

**Figure:**
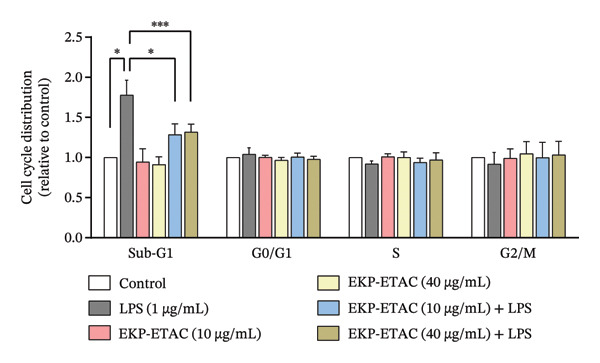
(b)

### 3.9. Effect of EKP‐ETAC on Suppressive Intracellular ROS

**FIGURE 7:**
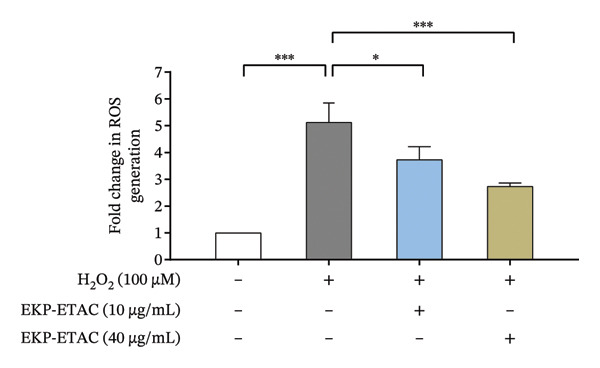
Intracellular ROS levels in HTR‐8/SVneo cells. Cells were preincubated with EKP‐ETAC and then stimulated with H_2_O_2_. Fluorescence intensity was detected at 1 h after stimulation. ^∗^ *p* < 0.05, ^∗∗∗^ *p* < 0.001.

We tested whether EKP‐ETAC could reduce the harmful effects of ROS when HTR‐8/SVneo cells were exposed to H_2_O_2_. The results demonstrated that H_2_O_2_ treated cells significantly elevated intracellular ROS levels (Figure 7). However, pretreatment with EKP‐ETAC at either 10 or 40 μg/mL significantly and dose‐dependently reduced ROS levels compared to the H_2_O_2_‐treated group. This evidence indicates that EKP‐ETAC has potent antioxidative properties, suppressing intracellular ROS production induced by H_2_O_2_ exposure.

### 3.10. Effect of EKP‐ETAC on miR‐138‐5p Expression in LPS‐Induced HTR‐8/SVneo Cells

**FIGURE 8:**
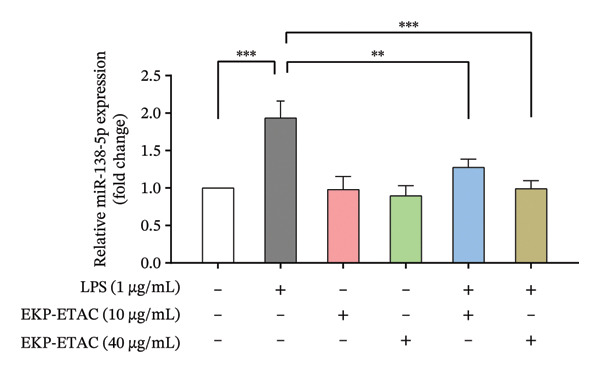
miR‐138‐5p expression in HTR‐8/SVneo cells. Cells were preincubated with EKP‐ETAC and then stimulated with LPS or treated with LPS and EKP‐ETAC alone for 24 h. Following this, miR‐138‐5p expression was determined using qRT‐PCR. ^∗∗^ *p* < 0.01, ^∗∗∗^ *p* < 0.001.

As miR‐138‐5p is recognized as an inflammatory miRNA, we hypothesized that EKP‐ETAC could counteract the elevated expression of miR‐138‐5p caused by LPS exposure in HTR‐8/SVneo cells. As demonstrated in Figure 8, the experiment revealed that LPS treatment significantly increased miR‐138‐5p expression compared to the untreated control. However, EKP‐ETAC pretreatment normalized miR‐138‐5p expression effectively and in a dose‐dependent manner. Notably, EKP‐ETAC treatment alone maintained baseline miR‐138‐5p expression. This evidence suggests that EKP‐ETAC mitigates the inflammatory response by regulating miR‐138‐5p expression patterns in trophoblast cells under LPS‐induced inflammatory conditions.

### 3.11. Potential Target of miR‐138‐5p and Validation

Bioinformatic analysis identified several potential targets of miR‐138‐5p, including SIRT1, DAPK2, CCAR2, FOXC1, and FOXP4. Among these, TargetScan predicted FOXC1 as a candidate, with a conserved binding site in its 3′‐UTR (positions 160–167) (Figure 9(a)).

FIGURE 9FOXC1 as a potential target of miR‐138‐5p. The mRNA level of FOXC1 was evaluated using qRT‐PCR in EKP‐ETAC pretreated HTR‐8/SVneo cells and then stimulated with LPS or treated with LPS and EKP‐ETAC alone for 24 h. The binding site for miR‐138‐5p on F0XC1 3′‐UTR (a) and FOXC1 mRNA expression in HTR‐8/SVneo cells (b). ^∗^ *p* < 0.05, ^∗∗^ *p* < 0.01, ^∗∗∗^ *p* < 0.001.

**Figure:**
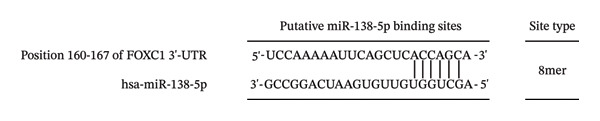
(a)

**Figure:**
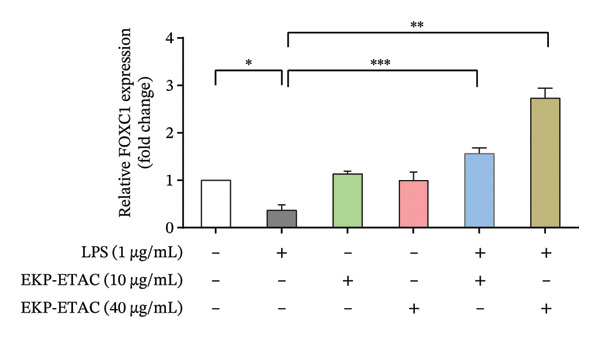
(b)

To investigate whether miR‐138‐5p regulates FOXC1 in HTR‐8/SVneo trophoblast cells, we first measured FOXC1 mRNA levels under inflammatory conditions. FOXC1 expression was significantly reduced in LPS‐treated cells compared to controls (Figure 9(b)), mirroring the LPS‐induced increase in miR‐138‐5p (Figure 8). Notably, pretreatment with EKP‐ETAC restored FOXC1 expression in LPS‐exposed cells, suggesting that EKP‐ETAC alleviates inflammation by modulating the miR‐138‐5p/FOXC1 axis.

To confirm direct targeting, we transfected cells with miR‐138‐5p mimic or NC mimic. Overexpression of miR‐138‐5p significantly downregulated FOXC1 mRNA, whereas the NC mimic had no effect (Figure 10(a)). We then constructed luciferase reporters containing either the WT or MUT miR‐138‐5p binding site from the FOXC1 3′‐UTR (Figure 10(b)). Co‐transfection with miR‐138‐5p mimic significantly suppressed luciferase activity in cells carrying the WT reporter, but not the MUT reporter (Figure 10(c)). No reduction was observed with the NC mimic in either construct.

FIGURE 10Confirmation of FOXC1 as a direct target of miR‐138‐5p. FOXC1 mRNA expression levels in HTR‐8/SVneo cells transfected with negative control (NC) mimic or miR‐138‐5p mimic were determined using qRT‐PCR (a). Sequences of wild‐type (WT) and mutant (MUT) FOXC1 3′‐UTR used for luciferase reporter assays (b). Luciferase activity in cells cotransfected with FOXC1 3′‐UTR‐WT or FOXC1 3′‐UTR‐MUT luciferase reporter plasmids, along with NC mimic or miR‐138‐5p mimic, for 48 h (c). ^∗∗^ *p* < 0.01, ^∗∗∗^ *p* < 0.001.

**Figure:**
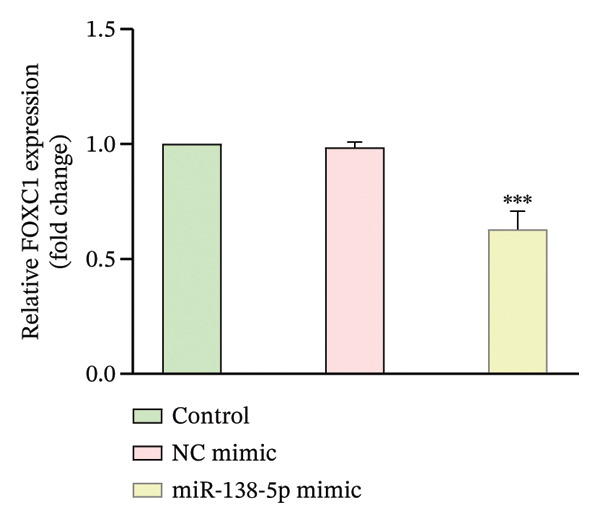
(a)

**Figure:**
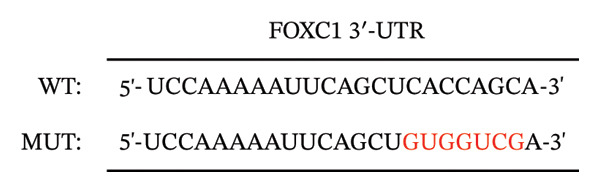
(b)

**Figure:**
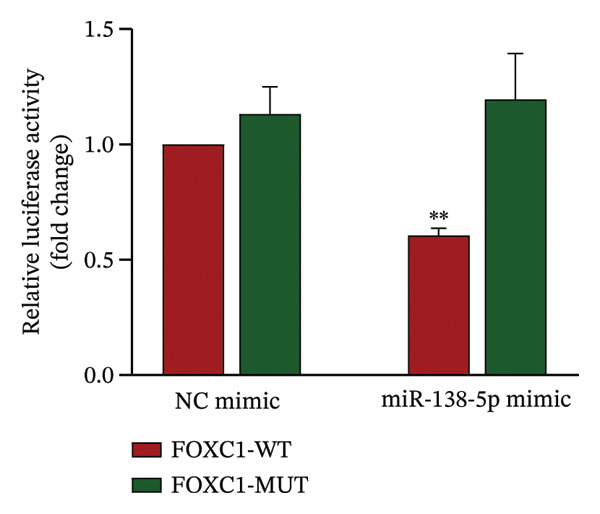
(c)

These results provide direct evidence that FOXC1 is a bona fide target of miR‐138‐5p. The LPS‐induced downregulation of FOXC1 is mediated, at least in part, by miR‐138‐5p‐dependent post‐transcriptional repression. Moreover, our findings demonstrate that EKP‐ETAC protects trophoblast cells from inflammatory injury by suppressing miR‐138‐5p and restoring FOXC1 expression.

### 3.12. Identification of Bioactive Compounds in EKP‐ETAC

The high phytochemical content and potent biological activities of EKP‐ETAC prompted the identification of its bioactive compounds using LC–MS analysis. The results revealed eight major compounds with retention times of 6.354, 14.503, 15.805, 16.134, 16.261, 16.664, 17.729, and 20.768 min, respectively. Each compound was fragmented, producing spectra with candidate masses (*m/z*) of 1034.024, 447.107, 515.139, 359.088, 719.185, 467.228, 313.080, and 1034.024, respectively. Using an internal spectral database, the compounds were identified as caffeic acid, kaempferol 3‐O‐rutinoside, 5,6‐dihydroxy‐3′,4′,7,8‐tetramethoxyflavone, rosmarinic acid, vanillin, apigenin, benzaldehyde, and linoleic acid (Table 5). Further analysis of EKP‐ETAC was performed using HPLC with standards for chlorogenic acid, vanillin, rosmarinic acid, and luteolin. The HPLC chromatogram revealed that the highest peak corresponded to rosmarinic acid, with a concentration of 2083 μg/mL (Figures 11(a), 11(b)). This concentration was notably higher than those of chlorogenic acid, vanillin, and luteolin. These findings identify rosmarinic acid as the primary bioactive compound in EKP‐ETAC, which may play a significant role in its observed biological activities.

**TABLE 5:** LC–MS phytochemical profiling in EKP‐ETAC.

No.	RT (min)	Mass (Da)	*m/z*	Formula	Molecules	DB diff (ppm)
1	6.354	180.042	1034.024	C_9_H_8_O_4_	Caffeic acid	1
2	14.503	594.159	447.107	C_2_H_30_O_15_	Kaempferol 3‐O‐rutinoside	−0.03
3	15.805	74.100	515.139	C_19_H_18_O_8_	5, 6‐Dihydroxy‐3′, 4′, 7, 8‐tetramethoxyflavone	0.15
4	16.134	360.085	359.088	C_18_H_16_O	Rosmarinic acid	1.4
5	16.261	152.048	719.185	C_8_H_8_O_3_	Vanillin	1.15
6	16.664	270.053	467.228	C_15_H_10_O_5_	Apigenin	1.82
7	17.729	106.042	313.080	C_7_H_6_O	Benzaldehyde	−3.08
8	20.768	280.241	1034.024	C_18_H_32_O_2_	Linoleic acid	1.09

FIGURE 11HPLC chromatogram of standards (a) and EKP‐ETAC (b).

**Figure:**
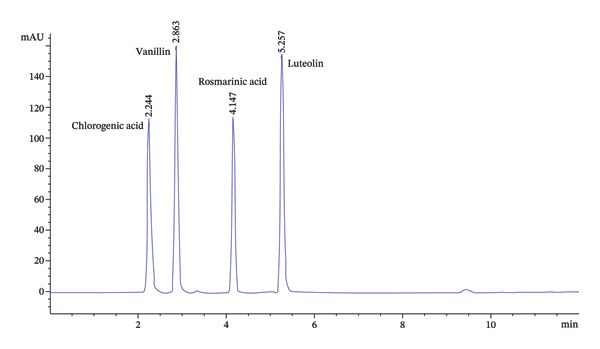
(a)

**Figure:**
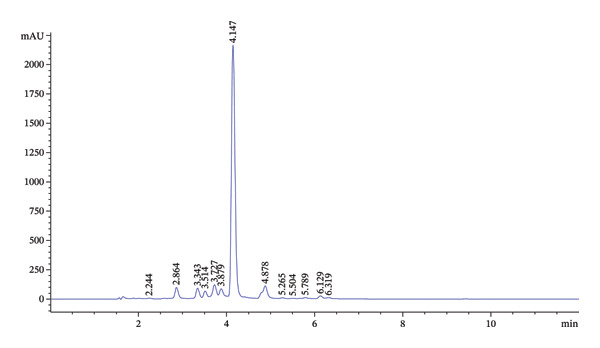
(b)

## 4. Discussion

The genus *Elsholtzia* (Lamiaceae family) includes species traditionally used in culinary practices and traditional medicine by ethnic communities in northern Thailand [21]. Among these, EKP holds notable cultural and ethnobotanical significance for local tribal groups. Despite its importance, limited research has been conducted on its phytochemical properties and biological activities.

This study investigated the biological activities of EKP, particularly focusing on its ETAC extract fraction (EKP‐ETAC). Among all fractions tested, EKP‐ETAC contained the highest concentrations of phenolic compounds and flavonoids. The extract demonstrated antioxidant capabilities equivalent to those of well‐established antioxidants ascorbic acid and Trolox. These findings position EKP‐ETAC as a promising natural antioxidant.

Notably, this research is the first to reveal the protective effects of EKP‐ETAC on HTR‐8/SVneo trophoblast cell functions under LPS‐induced inflammatory conditions. Pregnancy‐related inflammation, triggered by microbial and nonmicrobial factors, is a major contributor to complications such as preterm birth, miscarriage, preeclampsia, and fetal developmental disorders [22]. EKP‐ETAC mitigated these effects by reducing proinflammatory cytokine expression (IL‐1, IL‐6, and TNF‐α), alleviating oxidative stress, and preventing apoptosis in trophoblast cells. These findings suggest the potential of EKP‐ETAC to preserve trophoblast cell function, which is critical for successful placentation and a healthy pregnancy.

Trophoblast invasion, migration, proliferation, and apoptosis resistance are essential processes in placental development. Dysregulation of these processes is associated with complications such as placenta previa, pregnancy loss, fetal growth restriction (FGR), and preeclampsia [23]. The ability of EKP‐ETAC to support these functions underscores its potential role in promoting maternal and fetal health.

miRNAs are key regulators of inflammation. Proinflammatory miRNAs, including miR‐34a, miR‐34b, miR‐29c, miR‐132, miR‐138, miR‐155, and miR‐200, play critical roles in inflammatory responses [24]. A number of miRNAs have been implicated in the response to oxidative stress, potentially influencing redox balance [25–27]. It is well recognized that LPS stimulates intracellular ROS generation [28]. Previous studies have shown that LPS exposure increases miR‐138‐5p expression in different cell lines [29]. In addition, the overexpression of miR‐138 led to a decrease in the proliferation and migration of HTR‐8/SVneo cells [10]. This study demonstrated that EKP‐ETAC modulates miR‐138‐5p expression, effectively restoring trophoblast functionality. EKP‐ETAC pretreatment suppressed LPS‐induced upregulation of miR‐138‐5p in HTR‐8/SVneo trophoblast cells, highlighting its potential as a therapeutic agent.

Further analysis identified FOXC1 as a potential target of miR‐138‐5p. FOXC1 expression inversely correlated with miR‐138‐5p levels, with FOXC1 playing an essential role in embryonic and organ development [30]. FOXC1 overexpression has been linked to reduced apoptosis and increased cell proliferation [31], while FOXC1 silencing produces opposite effects [32]. FOXC1 also exhibits anti‐inflammatory properties, including suppression of IL‐1β in LPS‐induced breast cancer cells and protecting HTR‐8/SVneo trophoblast cells from apoptosis under high‐glucose conditions [33]. This study found that FOXC1 is the direct target of miR‐138‐5p and EKP‐ETAC restored FOXC1 expression, supporting its role in enhancing cell proliferation, reducing apoptosis, and mitigating inflammation in trophoblast cells.

The growing interest in natural bioactive compounds for maternal health reflects a broader shift toward integrative medicine. Many of these compounds exhibit anti‐inflammatory, antioxidant, antihypertensive, and immunomodulatory properties that directly address pathophysiological mechanisms underlying adverse pregnancy outcomes such as preeclampsia, GDM, preterm labor, and intrauterine growth restriction (IUGR). Several such compounds have already advanced from experimental models to clinical use or are currently under clinical evaluation, highlighting their translational potential.

Resveratrol, a polyphenolic stilbene found in grapes, berries, and red wine, is among the most extensively studied natural compounds in this context. Preclinical studies have consistently demonstrated its ability to reduce oxidative stress, suppress proinflammatory cytokines, and improve endothelial function. These activities position resveratrol as a potential therapeutic agent for pregnancy‐related disorders, including insulin resistance, GDM, dyslipidemia, preeclampsia, and FGR [34].

Curcumin, the active polyphenol in turmeric, exhibits well‐documented antioxidant and anti‐inflammatory effects and has been proposed for managing FGR, preterm birth, GDM, and preeclampsia [35, 36]. A clinical study in preeclampsia patients reported that curcumin significantly reduced cyclooxygenase‐2 (COX‐2) levels, Visual Analog Scale (VAS) scores, and anticoagulant factors, suggesting analgesic, anti‐inflammatory, and anticoagulant benefits [37]. These findings support curcumin’s therapeutic potential in pregnancy‐related complications, though further research is needed to establish optimal dosing and treatment protocols.

Quercetin, a dietary flavonoid abundant in fruits, vegetables, seeds, and nuts [38], has recently been shown to exert antioxidant effects in trophoblast cells by upregulating HO‐1 and NRF2 during syncytialization, a process associated with ROS generation [39]. In vivo, quercetin prevented embryo loss in a spontaneous abortion model [40], suggesting its potential to alleviate pregnancy complications.

Bioactive compound analysis identified rosmarinic acid as the primary constituent of EKP‐ETAC, along with minor components including chlorogenic acid, vanillin, and luteolin. Rosmarinic acid, a phenolic compound abundant in the Lamiaceae and Boraginaceae families, is well‐known for its antioxidant, anti‐inflammatory, antibacterial, anticancer, and antidiabetic properties [41]. Vanillin and luteolin also exhibit anti‐inflammatory effects and mitigate oxidative stress in both in vitro and in vivo models [42]. The pharmacological activity of EKP‐ETAC likely results from the synergistic action of these primary and secondary bioactive compounds.

This study highlights EKP‐ETAC as a promising natural agent for addressing pregnancy‐related complications associated with inflammation and oxidative stress. Its potential applications extend to other conditions driven by chronic inflammation and oxidative stress.

## 5. Conclusions

In conclusion, this study highlights the remarkable therapeutic potential of EKP‐ETAC, particularly in managing inflammation‐related complications in pregnancy. By demonstrating potent antioxidant and anti‐inflammatory properties, EKP‐ETAC effectively modulates critical molecular pathways, including the downregulation of miR‐138‐5p and restoration of FOXC1 expression, to support trophoblast function. Its ability to reduce oxidative stress, suppress proinflammatory cytokines, and enhance trophoblast proliferation, migration, and invasion positions EKP‐ETAC as a promising natural therapeutic agent. These findings provide a strong foundation for further exploration of EKP and its bioactive constituents in clinical applications targeting pregnancy complications and other inflammation‐driven conditions.

## Author Contributions

Wittaya Chaiwangyen: conceptualization; data curation; formal analysis; funding acquisition; investigation; methodology; project administration; resources; supervision; validation; visualization; writing–original draft; and writing–review and editing. Orawan Khantamat: conceptualization; data curation; methodology; validation; and writing–review and editing. Amnart Onsa‐ard, Napapan Kangwan, and Wachiraporn Tipsuwan: conceptualization; investigation; methodology; validation; and writing–review and editing. Angkana Songkrao, Apirak Katasai, Apisit Phngam, Piyawan Nuntaboon, Ramita Sangprachum, and Pilaiporn Thippraphan: formal analysis and investigation. Francisco Lázaro Pereira de Sousa: conceptualization; formal analysis; methodology; and validation.

## Funding

This study was supported by the University of Phayao and Thailand Science Research and Innovation Fund (Fundamental Fund 2024); National Research Council of Thailand (NRCT) and University of Phayao, N42A660433; Plant Genetic Conservation Project Under the Royal Initiative of Her Royal Highness Princess Maha Chakri Sirindhorn Conducted by University of Phayao, RSPG670030.

## Conflicts of Interest

The authors declare no conflicts of interest.

## Data Availability

The data that support the findings of this study are available from the corresponding author upon reasonable request.
